# Regulation of X-Ray Irradiation on the Activity and Expression Levels of CYP1A2 and CYP2E1 in Rats

**DOI:** 10.3389/fphar.2019.01575

**Published:** 2020-01-28

**Authors:** Xiang-Yang Li, Ning Qu, Xue-Jun Wang, Jian-Xin Yang, Yuan-Yao Xin, Jun-Bo Zhu, Xue Bai, Ya-Bin Duan

**Affiliations:** ^1^ State Key Laboratory of Plateau Ecology and Agriculture, Qinghai University, Xining, China; ^2^ Medical College, Qinghai University, Xining, China; ^3^ Department of Anesthesiology, Qinghai Hospital of Traditional Chinese Medicine, Xining, China; ^4^ Department of Anesthesiology, Red Cross Hospital of Qinghai, Xining, China; ^5^ College of Eco-Environmental Engineering, Qinghai University, Xining, China

**Keywords:** CYP1A2, CYP2E1, X-ray irradiation, activity, expression, pharmacokinetics

## Abstract

The objective of this study was to investigate the regulation of X-ray irradiation and its effect on the activity and protein and mRNA expression levels of CYP1A2 and CYP2E1 in rats. Rats were randomly divided into 0 Gy (control), 1 Gy (low-dose irradiation), and 5 Gy (high-dose irradiation) groups. CYP1A2 and CYP2E1 activity was evaluated from changes in pharmacokinetic parameters of caffeine and chlorzoxazone, respectively. The plasma concentrations of the probe drugs were determined by high-performance liquid chromatography (HPLC). Enzyme-linked immunosorbent assay (ELISA) and real-time polymerase chain reaction (PCR) tests were used to analyze the protein and mRNA expression levels of CYP1A2 and CYP2E1, respectively. The AUC_0-12_ of caffeine was decreased by 1.7- and 2.5-fold, and the CL was increased by 1.8- and 2.6-fold in the 1 Gy and 5 Gy groups, respectively, compared to the 0 Gy group. The AUC_0-10_ of chlorzoxazone was 1.4- and 1.8-fold lower, and the CL was 1.4- and 1.9-fold higher in the 1 Gy and 5 Gy groups, respectively, compared to the 0 Gy group. The metabolism of caffeine and chlorzoxazone increased under X-ray irradiation as CL levels increased and AUC levels decreased, suggesting that CYP1A2 and CYP2E1 activity is enhanced in rats after X-ray irradiation. Compared to that of the 0 Gy group, the protein expression level of CYP1A2 was measured as 28.3% and 38.9% higher in the 1 Gy and 5 Gy groups, respectively. The protein expression level of CYP2E1 was 48.4% higher in the 5 Gy group compared to the 0 Gy group, and there was no statistically significant difference between 0 Gy and 1 Gy. Compared to the 0 Gy group, the mRNA expression level of CYP1A2 was 200% and 856.3% higher in the 1 Gy and 5 Gy group, respectively, whereas the mRNA expression level of CYP2E1 was 89.0% and 192.3% higher in the 1 Gy and 5 Gy groups, respectively. This study reveals significant changes in the activity and protein and mRNA expression levels of CYP1A2 and CYP2E1 in rats after exposure to X-ray irradiation.

## Introduction

Cytochrome P450 enzyme-based drug metabolism is dependent on oxygen. Interindividual differences in P450 isozyme activity are attributable to genetic polymorphisms and specific xenobiotic induction or inhibition (Eichelbaum et al., 1992; Guengerich, 1995). To date, our understanding of the role of nonchemical factors, including biophysical influences, disorders, pathological states, and various constitutional properties of interindividual variations in hepatic drug metabolism, remains limited (Jones et al., 1989; Vesell, 1997). Irradiation is considered to be one of the oldest and most common pathogenic factors (Li et al., 2019).

Irradiation pertains to the process of distributing energy into space or matter using waves and particles (Repacholi, 2017; Li et al., 2019). Ionizing and non-ionizing irradiation have been extensively utilized in medicine, agriculture, food processing, national defense, and daily activities, and they have also negatively influenced human health (Sato and Zouain, 2012; Sutlief, 2015; Andrianov et al., 2015; von Neubeck et al., 2015). Ionizing irradiation includes X-ray and γ-ray. A γ-ray source is typically cesium-137 or cobalt-60. X-rays are stimulated by the inner electrons of the atoms, while γ-rays come from the nucleus. Compared to γ-rays, X-ray irradiation units are less expensive and have no radioactive source. X-ray irradiation is a possible alternative to γ-ray irradiation in clinical settings due to its reliability, wide energy wavelength, and user-friendly interface *(* Janatpour et al., 2005; Thwaites and Tuohy, 2006 *)*. Scientists have uncovered a series of physiological variations and pathological divergences induced by irradiation, and these influence drug absorption, distribution, metabolism, and excretion from the body (Rendic and Guengerich, 2012; Qiao et al., 2017). Exposure to irradiation has significantly increased with the use of nuclear power and technology in various industries (e.g., agriculture and medicine). Currently, individuals susceptible to irradiation exposure include those working in radioactive areas and residents living in high-altitude zones. Therefore, it is essential for medical researchers to rationalize drug use for these specific populations. Additional changes in drug metabolism observed after irradiation have been reported with an increasing amount of cancer patients treated conventionally with radiotherapy methods (Rendic and Guengerich, 2010; Rendic and Guengerich, 2012; Qiao et al., 2017).

Investigations into preventing and treating irradiation damage, drug efficacy, and mitigating the effects of irradiation on the pharmacokinetics of drugs, such as 5-fluorouracil, cisplatin, and irinotecan hydrochloride, have been conducted for years (Hsieh et al., 2011; Duan et al., 2015; Tsai et al., 2015; Spetz et al., 2018; Qiao et al., 2019). These studies have observed significant differences in the characteristics of drugs in experimental rats after irradiation exposure. Physiological changes observed after irradiation exposure may change drug pharmacokinetics that may require modifications in dosage regimens to maintain efficacy and/or prevent toxicity. Cytochrome P450 enzymes play a role in not only the phase I-dependent metabolism of xenobiotics but also in the metabolism of endogenous compounds. The CYP1A2 and CYP2E1 phase I enzymes are responsible for the metabolic activation and detoxification of several chemical compounds. Caffeine and chlorzoxazone have been widely regarded as probe substrates used to evaluate the activity of CYP1A2 and CYP2E1, respectively, *in vivo* (Yong et al., 2016; Satish and Prasad, 2018). Earlier studies have reported an increase in CYP1A2, CYP2B1, CYP2C9, CYP2E1, and CYP3A1 mRNA and protein levels in rats after γ-ray irradiation exposure (Chung et al., 2001; Maksymchuk et al., 2008; Yi et al., 2015).

Investigations into changes in the activity and expression of cytochrome P450 due to X-ray irradiation are scarce. The effects of X-ray irradiation exposure on CYP3A1 and carboxylesterase (CES) expression in rats have recently been investigated by our team (Marchenko et al., 2010; Qiao et al., 2019). However, no reports on changes in CYP1A2 and CYP2E1 expression levels and activity due to X-ray irradiation have been published to date. The present study investigated changes in the activities and mRNA and protein expression levels of CYP1A2 and CYP2E1 in rats after X-ray irradiation at doses of 1 Gy and 5 Gy with the goal of providing information about rational drug use during concurrent chemoradiation therapy.

## Materials and Methods

### Chemicals, Reference Standards, and Solvents

Chlorzoxazone (lot: LC90O72) was purchased from J&K Scientific Corporation (Beijing, China). Caffeine (lot: 20071224) was obtained from Sinopharm Chemical Reagent Co., Ltd. (Shanghai, China). Rat CYP1A2 and CYP2E1 ELISA Kits (lot: 20180511, 20180321) were purchased from Fangcheng Beijing Technology Co., Ltd. (Beijing, China). Biochemical reagents kits were purchased from Jiangxi Tekang Technology Co., Ltd.: alanine phosphatases (lot: 1707110), transaminases GPT (lot: 1707136), transaminases GOT (lot: 1707132), total bilirubin (lot: 1707113), direct bilirubin (lot: 1707128), indirect bilirubin (lot: 1707126), total protein (lot: 1707131), albumin (lot: 1707127), globulin (lot: 1707108), creatinine assay kit (lot: 1707129), and uric acid (lot: 1707125). HPLC-grade methanol (lot: 20170507) was purchased from Shandong Yuwang Company Inc. (Jinan, China). All other chemicals and solvents were obtained from commercial sources at the highest grade available. HEPES were obtained from AMRESCO Company Inc. (Boise, USA). RNAiso Plus (lot: 9109), Prime-Script™ RT Reagent (Lot: K1622), and SYBR premix EX Taq™ II (lot:AK6401) were obtained from Takara (Kyoto, Japan). Primers used for real-time PCR were synthesized by Takara.

### Animals

Sprague Dawley SPF rats (200 ± 20 g, certificate No. 2007-001) of both sexes were provided by the laboratory animal center at Xi'an Jiaotong University Medical College, China. They were adapted for a week at 23 ± 2°C with a constant humidity level of 55% ± 5% under a cycle of 12 h of dark conditions and given *ad libitum* access to water and food pellets. Three animals were housed per cage in separate rooms to ensure that each animal was restrained to a single space to prevent the development of restraint stress. All experimental procedures were applied in strict accordance with the National Institutes of Health Guide for the Care and Use of Laboratory Animals.

Thirty clean SD rats were randomly divided into 0 Gy (control), 1 Gy (low dose irradiation), and 5 Gy (high dose irradiation) groups, and every group included five male and five female rats. All rats, except for those in the control group, were restrained in special boxes and exposed to 1 or 5 Gy whole-body X-ray irradiation from a medical electronic linear accelerator (23EX, Varian, Palo Alto, CA, USA). We chose plastic bottles to make a mold that fit the shape of the rats to restrain the rats in the chamber (**Supplementary Figure S1**). The upper end of the bottle was small and the lower end was sealed to limit activity whilst also ensuring the breathing of the rats. The same group of rats was neatly arranged in the center of the electron accelerator field of view before irradiation. The source-to-animal distance was set as 100 cm, and the dose rate was set to 300 cGy/min. The irradiation period was set to 20 s and 100 s for the 1 Gy and 5 Gy doses, respectively. We applied 1 and 5 Gy to the rats to simulate the relevant dosage range for the daily treatment of a human torso and in the interest of safety and workability, as described in our previous report (Duan et al., 2015; Li et al., 2019; Qiao et al., 2019).

### Determination of Physiologic and Blood Parameters

A routine blood examination was performed with an XFA6100 automatic hematology analyzer (Nanjing Perlove Medical Equipment, Co., Ltd., China), and blood biochemical parameters were determined by applying an AU2700 automatic biochemistry analyzer (Olympus Corporation, Japan) to rats after exposure to X-ray irradiation.

### Determination of Thymus and Spleen Indexes

The thymus and spleen were removed from each rat 40 h after X-ray irradiation. Thymus and spleen indexes were calculated by dividing the organ weight by the bodyweight (BW):

Thymusindex(%)=thymusweight (g) / BW (g)×100%

Spleenindex(%)=spleenweight (g) / BW (g)×100%

### Measurement of Rat CYP1A2 and CYP2E1 Activity

CYP1A2 and CYP2E1 activities in rats were identified using the previously reported “cocktail” method (Li et al., 2014a; Li et al., 2014b). Caffeine and chlorzoxazone served as probe substrates of CYP1A2 and CYP2E1, respectively. The activities of CYP1A2 and CYP2E1 were evaluated by the pharmacokinetic characteristics of caffeine and chlorzoxazone, respectively. The rats in the 1 Gy or 5 Gy groups were given probe drugs 40 hours after receiving X-ray irradiation. To evaluate the effect of X-ray irradiation on CYP1A2 activity, rats from three groups were given a caffeine solution orally at a dose of 60 mg/kg after an overnight fast of ≥ 12 h (with water allowed ad libitum) (Wang et al., 2009). A total of 0.3 ml of serial blood samples taken from the eye socket was collected before (baseline) the study drug was given and 0.083, 0.167, 0.333, 0.5, 1, 2, 4, 6, 8, and 12 h after study drug administration. To evaluate the effect of X-ray irradiation on CYP2E1 activity, rats from three groups were given a chlorzoxazone solution orally at a dose of 100 mg/kg after an overnight fast of ≥12 h (with water allowed ad libitum) (Yang, 2014). A total of 0.3 ml of serial blood samples taken from the eye socket was collected before (baseline) the study drug was given and 0.083, 0.167, 0.333, 0.5, 1, 2, 4, 6, 8, and 10 h after study drug administration. Plasma samples were separated by centrifuging the blood samples at 3,000 g for 10 min at 4°C and were immediately stored at -20°C until the quantitative drug analysis.

### Bioanalytical Methods

The plasma concentrations of caffeine and chlorzoxazone were determined by HPLC. A 0.12-ml aliquot was added to 0.04 ml of 30% perchloric acid in a 1-ml centrifuge tube. The tube was vortexed for 0.5 min and then centrifuged for 10 min at 16,000 g at 4°C. The HPLC system was then injected with 20 μL aliquot of supernatant.

HPLC analyses of caffeine and chlorzoxazone were performed using an HPLC pump (G7104C, Agilent Technologies, Inc., California, USA) and a diode array detection system (G7115A, Agilent Technologies, Inc., California, USA) equipped with a C_18_ column (Boston, Boston Analytics, Inc., Boston, USA; 4.6 × 250 mm, inner diameter, 10 μm). The mobile phase of caffeine was methyl alcohol-water (60∶40, v/v), and the column temperature was maintained at 25°C. A constant flow rate of 1.0 ml/min was employed throughout the analyses, and the detection was performed at 275 nm. The method was linear in the range of 0.5−60 μg/ml, and the correlation coefficients of the regression lines were always > 0.9995. The lower limit of quantitation was 0.5 μg/ml. The batch-to-batch CV of the spiked quality-control samples was 4.36%, 2.56%, 6.71%, and 3.85% for caffeine concentrations of 0.5, 1, 25, and 50 μg/ml, respectively. The accuracy levels were calculated as the difference between the given and measured mean concentrations expressed as a percentage, and they were measured as 5.83%, 5.67%, 3.11%, and 1.48%, respectively, at the aforementioned target concentrations. The mobile phase of chlorzoxazone was methyl alcohol-water (62∶38, v/v), and the column temperature was maintained at 25°C. A constant flow rate of 1.0 ml/min was employed throughout the analyses, and detection was performed at 287 nm. The method applied was linear in the range of 0.5−60 μg/ml, and the correlation coefficients of the regression lines were always > 0.9995. The lower limit of quantitation was 0.5 μg/ml. The batch-to-batch CV of the spiked quality-control samples was 6.50%, 2.28%, 1.70%, and 2.35% for chlorzoxazone concentrations of 0.5, 1, 25, and 50 μg/ml, respectively. The accuracy levels were calculated as the difference between the given and measured mean concentrations expressed as a percentage, and they were measured as -4.19%, -1.80%, -0.58%, and -1.78%, respectively, at the aforementioned target concentrations.

### Pharmacokinetic Analysis

Pharmacokinetic values for *caffeine and chlorzoxazone* were calculated for each rat, and these were used to determine mean values for analysis. A noncompartmental analysis using DAS 2.0 software (Institute of Clinical Pharmacology, Shanghai University of Traditional Chinese Medicine, Shanghai, China) was performed to calculate the elimination rate constant (K_e_), plasma t_1/2_, mean residence time (MRT), volume of distribution (V_d_), clearance (CL), and AUC_0–t_ and AUC_0–∞_. The values of T_max_ and C_max_ were directly obtained from the raw data.

### Preparation of Hepatic Microsomes

Ten rats of every group were decapitated after collecting the last blood sample. Liver tissues were collected and immediately frozen in liquid nitrogen. Liver microsomes were prepared *via* differential centrifugation according to our previous reports (Li et al., 2014a; Li et al., 2014b; Qiao et al., 2019). Briefly, after the liver samples were thawed and weighed, two volumes of ice-cold Tris-HCl buffer at pH 7.4 containing 0.25 M sucrose (50 mmol/L) were added. Liver tissue was cut using scissors and homogenized with an automatic homogenizer at 500 g (IKA T8, Labortechnik, Stanfen, Germany). The resulting homogenates were then transferred to clean centrifuge tubes and centrifuged at 10,000 g for 30 min at 4°C using a TGL-16B centrifuge (Anting Scientific Instrument Factory, Shanghai, China). The supernatant was then collected and centrifuged at 100,000 g for 80 min at 4°C using an Optima MAX-XP ultracentrifuge (Beckman Coulter Inc., California, USA). The microsomal pellet was resuspended in homogenization medium. Hepatic microsomal suspensions (0.5 mL) were then aliquoted into Eppendorf tubes and stored at -80°C.

### ELISA Analysis of CYP1A2 and CYP2E1 Proteins

The expression levels of CYP1A2 and CYP2E1 were measured using the ELISA method according to our previous reports (Li et al., 2014a; Li et al., 2014b). Briefly, to prepare a bland well, 50 μL of the sample and 50 μL of standard diluted solution were added to the sample and standard well, respectively, with a biotin-conjugated antibody specific to CYP1A2 and CYP2E1. Then, Avidin conjugated to horseradish peroxidase (HRP) was added to each microplate well and incubated for 30 min at 37°C. After the complete removal of the liquid, 200 μL of wash buffer was added to each well, equilibrated for 10 s, and centrifuged. This process was repeated for a total of five rinsing cycles, and any remaining wash buffer was then removed by aspirating and decanting after the last wash. Then, 50 μL of substrate A and 50 μL of substrate B were added to each well, mixed thoroughly, and incubated for 30 min at 37°C. The enzyme–substrate reaction was terminated with the addition of a sulfuric acid solution, and the resulting change in color was spectrophotometrically measured at a wavelength of 450 nm. CYP1A2 and CYP2E1 concentrations in the samples were then determined by comparing the O.D. of the samples to the standard curve. The equation of the standard curve for CYP1A2 was C = 4.3222 × O.D. - 0.2394. The method was linear in the range of 0.312−20 ng/ml, and the correlation coefficients of the regression lines were always > 0.9990. The lower limit of quantitation was 0.118 ng/ml. The CV of intraday precision was less than 10%, and the CV of interday precision was less than 12% for low, middle, and high-level CYP1A2. The equation of the standard curve for CYP2E1 was C = 5.118 × O.D. - 0.1377. The method was linear in the range of 3.12−200 ng/ml, and the correlation coefficients of the regression lines were always > 0.9970. The lower limit of quantitation was 1.41 ng/ml. The CV of intraday precision was less than 10%, and the CV of interday precision was less than 12% for low, middle, and high-level CYP2E1.

### Real-Time PCR Analysis of CYP1A2 and CYP2E1 mRNA

The livers of ten rats from every group were excised immediately after the rates were killed. A small portion of liver from the left lobe was snap-frozen in liquid nitrogen and stored at -80°C before the extraction of total RNA. Approximately 100−200 mg of liver tissue was homogenized, and total RNA was isolated with the TRIzol reagent. The quality of the RNA solution was determined with a spectrophotometer. Primers for rat CYP1A2 and CYP2E1, including the reaction protocol, were designed by Major Bio-pharm Technology Co., Ltd. (Shanghai, China). All real-time PCRs were carried out using a SYBR^®^ Premix Ex Taq™ Kit (Takara) in accordance with the manufacturer's instructions. Amplification was performed in PCR capillaries on a Light Cycler 2.0 Real-Time Detection System (Roche, USA). The amplification of pre-denatured products was performed at 94°C for 60 s, followed by 45 cycles at 95°C for 30 s, 58°C for 30 s and 72°C for 30 s, 95°C for 10 s, 65°C for 45 s, and at 40°C for 60 s. Fold induction values were calculated using the value of 2^-ΔΔCt^ where ΔCt represents differences in cycle threshold numbers between the target gene and the control gene β-actin and where ΔΔ Ct represents the relative change in differences between the control and treatment groups. The RNA samples were of sufficient quality for RT-qPCR analysis. Protein-free and intact RNA was indicated by purity and integrity assessment of total RNA. RT-qPCR efficiency over all samples was calculated according to the Minimum Information for Publication of Quantitative Real-Time PCR Experiments (MIQE) guidelines. Primer specificity was confirmed by melting curve analysis. Specific amplification of target reference genes was confirmed by the observation of a specific peak in the melting curve analysis (**Supplementary Figures S2** and **S3**).

CYP1A2 primer sequence:

5'-TCGGTGGCTAATGTCATCGG-3' (forward primer)

5'-ACCGGAAAGAAGTCCACAGC-3' (reverse primer)

CYP2E1 primer sequence:

5'-TGGCTACAAGGCTGTCAAGG-3' (forward primer)

5'- AGGCTGGCCTTTGGTCTTTT -3' (reverse primer)

β-actin primer sequence:

5'- CAGGTCATCACTATCGGCAAT -3' (forward primer)

5'- TGGCATAGAGGTCTTTACGGA -3' (reverse primer)

## Data Analysis

The results are presented as the mean ± SD values. Statistical analysis was performed using SPSS version 23.0 (SPSS, IBM, Armonk, NY, USA). One-way analysis of variance (ANOVA) was used for multigroup comparisons, and the least significant difference (LSD) test was used for two-group comparison. Values of *P* < 0.05 were considered statistically significant.

## Results

### HPLC Method Validation

The retention times for caffeine and chlorzoxazone were measured as approximately 6.8 min and 7.8 min, respectively, under the present analytical conditions. The peaks of caffeine and chlorzoxazone were well-differentiated, and no endogenous interference in the rat plasma was observed. Strong levels of caffeine linearity were achieved within a range of 0.5−60 μg/ml with all coefficients of correlation exceeding 0.9995. Intraday precision and accuracy levels ranged from 2.56% to 6.71% and 1.48% to 5.83%, respectively. Interday precision and accuracy levels in the rat plasma ranged from 3.88% to 9.50% and from 1.59% to 9.30%, respectively. Strong levels of chlorzoxazone linearity were achieved within a range of 0.5−60 μg/ml with all coefficients of correlation exceeding 0.9995. Intraday precision and accuracy levels ranged from 1.70% to 6.50% and from -4.19% to -0.58%, respectively. Interday precision and accuracy levels in the rat plasma ranged from 2.20% to 5.16% and from -1.54% to 1.10%, respectively.

### Physiologic and Blood Parameters

**Figure 1:**
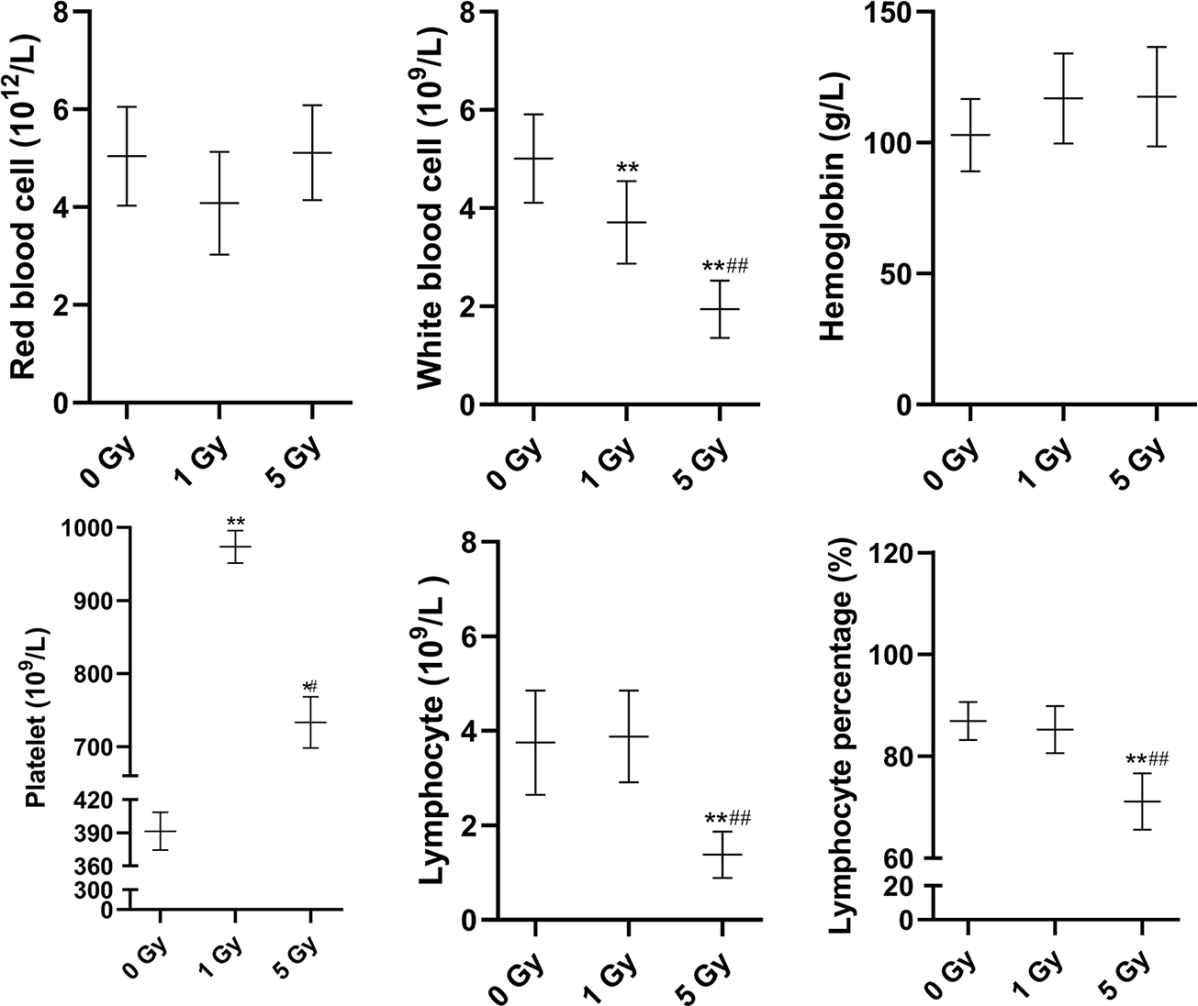
Changes in red blood cells, white blood cells, hemoglobin, platelets, lymphocytes, and lymphocyte percentage in the rats after X-ray irradiation. The data are presented as mean ± SD. N = 10. The data were analyzed using ANOVA, and the differences between the means of two groups were compared using LSD tests. ^*^ *P* < 0.05, ^**^ *P* < 0.01 compared to the 0 Gy group; ^#^ *P* < 0.05, ^##^ *P* < 0.01 compared to the 1 Gy group.

**Figure 2:**
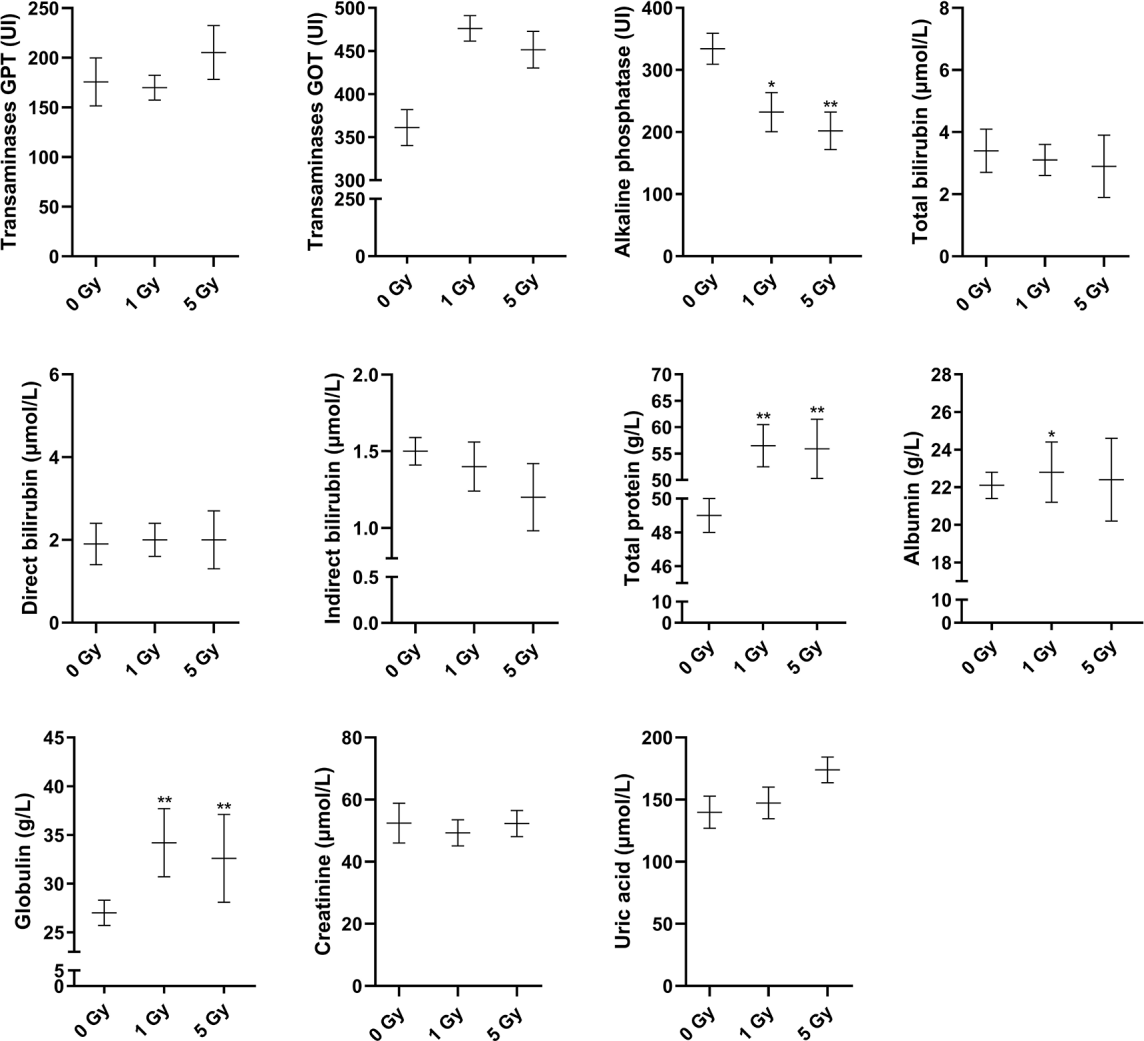
Changes in transaminases GPT, transaminases GOT, alkaline phosphatase, total bilirubin, direct bilirubin, indirect bilirubin, total protein, albumin, globulin, creatinine, and uric acid in the rats after X-ray irradiation. The data are presented as mean ± SD. N = 10. The data were analyzed using ANOVA, and the differences between the means of two groups were compared using LSD tests. *P < 0.05, **P < 0.01 compared to the 0 Gy group.

Changes in blood routine parameters induced by X-ray irradiation are shown in **Figure 1**, and the results of biochemical blood tests are shown in **Figure 2**. Compared to the 0 Gy group, the white blood cell count was 26.0% and 61.3% lower for the 1 Gy and 5 Gy groups (both *P* < 0.01), respectively, and 47.7% lower in the 5 Gy group than in the 1 Gy group (*P* < 0.01). The platelet count was 148.7% (*P* < 0.01) and 87.3% (*P* < 0.05) higher for the 1 Gy and 5 Gy groups, respectively, than for the 0 Gy group, and the count was 24.7% lower in the 5 Gy group than in the 1 Gy group (*P* < 0.05). Lymphocyte levels were 63.2% lower in the 5 Gy group than in the 0 Gy group (*P* < 0.01) and 64.4% lower in the 5 Gy group than in the 1 Gy group (*P* < 0.01). The lymphocyte percentage was 18.2% lower in the 5 Gy group than in the 0 Gy group (*P* < 0.01) and 16.6% lower in the 5 Gy group than in the 1 Gy group (*P* < 0.01). For red blood cells and hemoglobin, there was no significant difference when comparing the 1 Gy or 5 Gy groups with the 0 Gy group. Total protein levels were 15.3% and 14.0% higher for the 1 Gy and 5 Gy groups (both *P* < 0.01), respectively, than for the 0 Gy group. Compared to the 0 Gy group, globulin values were 26.7% and 20.7% higher for the 1 Gy and 5 Gy groups (both *P* < 0.01), respectively. Alkaline phosphatase levels were decreased by 30.5% (*P* < 0.05) and 39.6% (*P* < 0.01) in the 1 Gy and 5 Gy group, respectively, compared to the 0 Gy group. Albumin levels were 3.2% higher in the 1 Gy group than in the 0 Gy group (*P* < 0.05). For transaminases GPT, transaminases GOT, total bilirubin, direct bilirubin, indirect bilirubin, creatinine, and uric acid, and there was no significant difference when comparing the 1 Gy or 5 Gy groups with the 0 Gy.

### Thymus and Spleen Indexes

**Figure 3:**
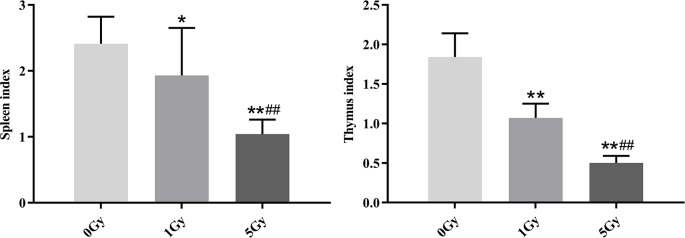
Thymus and spleen indexes in rats after exposure to X-ray irradiation. The data are presented as mean ± SD. N = 10. The data were analyzed using ANOVA, and the differences between the means of two groups were compared using LSD tests. *P < 0.05, **P < 0.01 compared to the 0 Gy group; ^##^ *P* < 0.01 compared to the 1 Gy group.

**Figure 3** shows the thymus and spleen indexes for the rats after X-ray irradiation. Thymus indexes were 41.8% and 72.8% lower in the 1 Gy and 5 Gy groups (both P < 0.01), respectively, compared to those in the 0 Gy group, and they were 53.3% lower in the 5 Gy group than in the 1 Gy group (P < 0.01). The spleen index was 19.9% (P < 0.05) lower in the 1 Gy group and 56.8% lower in the 5 Gy group (P < 0.01) than in the 0 Gy group, and it was 46.1% lower in the 5 Gy group than in the 1 Gy group (P < 0.01).

### Pharmacokinetics

**Figure 4:**
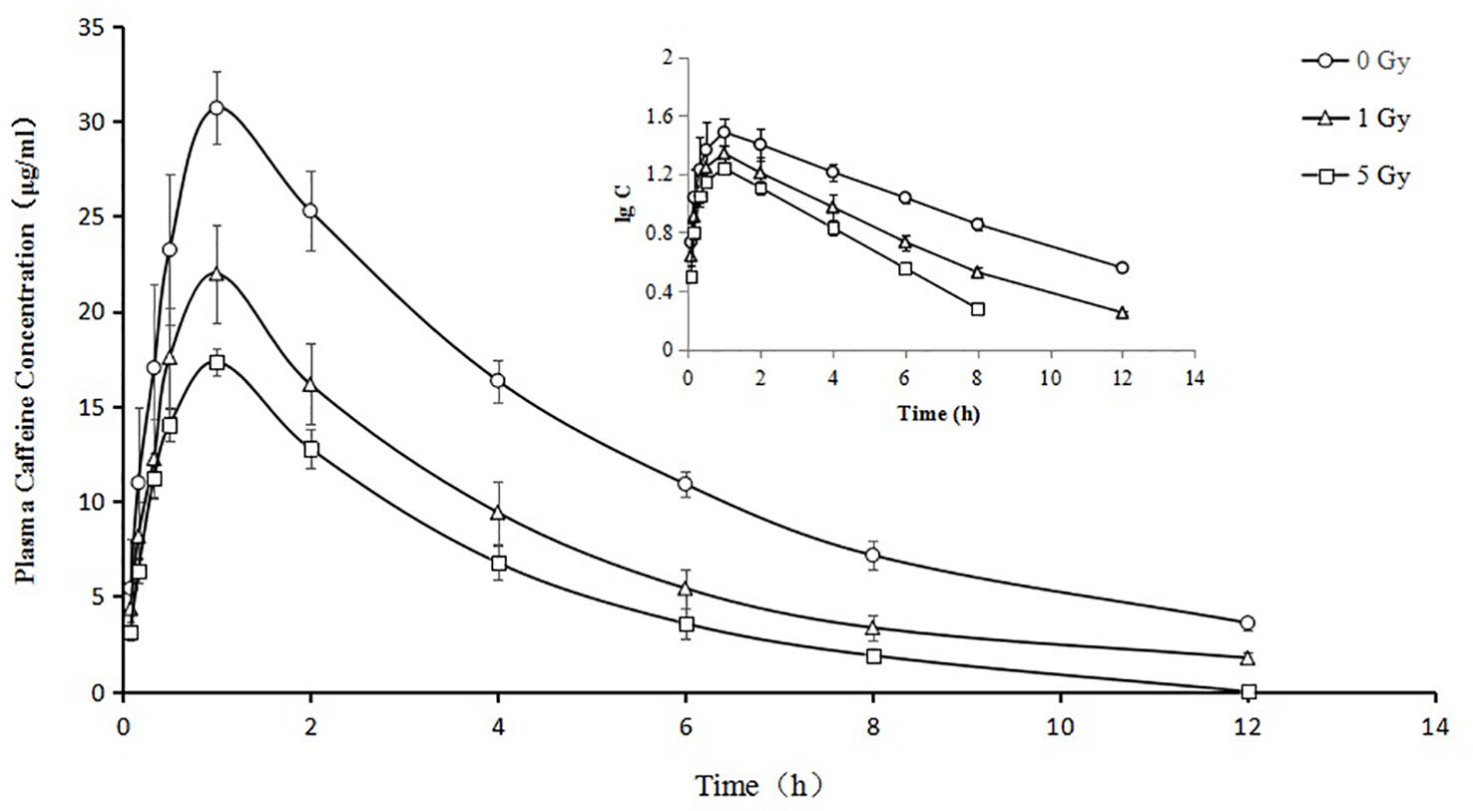
Mean plasma concentration time curve for oral caffeine (60 mg/kg) for rats subjected or not subjected to 1 Gy and 5 Gy of X-ray irradiation (N = 10).

**Figure 5:**
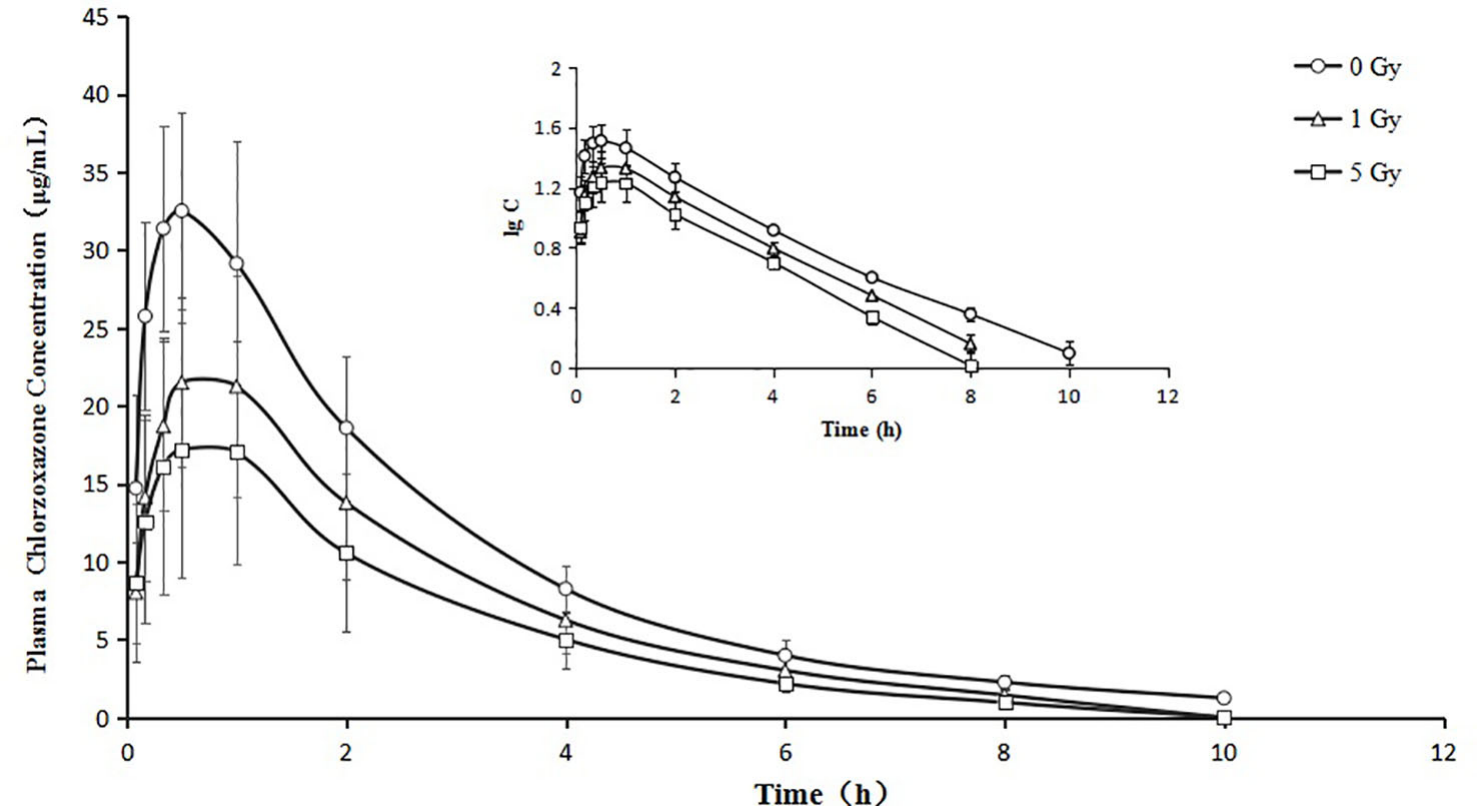
Mean plasma concentration time curve for oral chlorzoxazone (100 mg/kg) for rats subjected or not subjected to 1 Gy and 5 Gy of X-ray irradiation (N = 10).

The pharmacokinetic parameters of caffeine and chlorzoxazone were not significantly different between genders, and both data were combined. The mean pharmacokinetic parameters of caffeine and chlorzoxazone in the rat plasma are listed in **Tables 1** and **2**, respectively. **Figures 4** and **5** show mean plasma concentration–time profiles for caffeine and chlorzoxazone, respectively. Concentration–time profiles for *caffeine and chlorzoxazone* in plasma obtained for the three groups are similar in shape. Comparisons drawn between t_1/2_ in *caffeine* obtained from the rat plasma for the three groups studied revealed declines of 24.7% and 41.1% (both *P* < 0.01) after 1 and 5 Gy X-ray irradiation, respectively. Compared to the 0 Gy group, MRT_0-12_ values were 10.7% and 33.1% lower for the 1 Gy and 5 Gy groups (both *P* < 0.01), respectively. We also observed 1.7- and 2.5-fold declines for AUC_0-12_ in the 1 Gy and 5 Gy groups (both *P* < 0.01), respectively, compared to the 0 Gy group, and we observed a 1.5-fold decline in the 5 Gy group compared to the 1 Gy group (*P* < 0.01). CL values were 1.8- and 2.6-fold higher for the 1 Gy and 5 Gy groups (both *P* < 0.01), respectively, than for the 0 Gy group, and 1.4-fold higher in the 5 Gy group than in the 1 Gy group (*P* < 0.01). The V_d_ value was 38.8% and 51.9% (*P* < 0.01) higher in the 1 Gy and 5 Gy groups, respectively, compared with the 0 Gy group. The C_max_ value was 28.3% and 43.5% (*P* < 0.01) lower in the 1 Gy and 5 Gy groups, respectively, than in the 0 Gy group. For T_max_, there was no significant difference when comparing the 1 Gy or 5 Gy groups with the 0 Gy.

**Table 1:** Pharmacokinetic parameters of caffeine in rat plasma after X-ray irradiation.

Parameters	0 Gy	1 Gy	5 Gy
K_e_, 1/h	0.19 ± 0.02	0.25 ± 0.04^**^	0.32 ± 0.04^**##^
t_1/2_, h	3.72 ± 0.39	2.80 ± 0.53^**^	2.19 ± 0.26^**#^
MRT_(0-12)_, h	3.99 ± 0.15	3.56 ± 0.11^**^	2.67 ± 0.10^**##^
CL, L/kg/h	0.34 ± 0.02	0.62 ± 0.10^**^	0.89 ± 0.09^**##^
Vd, L/kg	1.83 ± 0.24	2.54 ± 0.04	2.78 ± 0.21^**^
AUC_(0-12)_, h·μg/ml	156.53 ± 10.07	93.32 ± 13.50^**^	62.36 ± 5.47^**##^
AUC_(0-∞)_, h·μg/ml	175.66 ± 9.22	98.64 ± 15.13^**^	68.52 ± 7.30^**##^
AUMC_(0-12)_, h^2^·μg/ml	624.18 ± 36.12	322.53 ± 52.37^**^	166.81 ± 20.26^**##^
AUMC_(0-∞)_, h^2^·μg/ml	964.39 ± 85.95	451.12 ± 85.56^**^	235.113 ± 39.92^**##^
T_max_, h	1.23 ± 0.35	1.35 ± 0.56	1.41 ± 0.21
C_max_, μg/ml	30.67 ± 1.98	21.98 ± 2.59	17.34 ± 0.74^**^

Data are expressed as the mean ± SD values (N = 10). *P < 0.05, **P < 0.01 compared to the 0 Gy group; ^#^ *P* < 0.05, ^##^ *P* < 0.01 compared to the 1 Gy group.

ke, elimination rate constant; t1/2, half-life; CL, total plasma clearance; Vd, apparent volume of distribution; Cmax, the maximum plasma concentration; AUC, area under the concentration time curve; AUMC, AUC of the first moment; MRT, mean residence time.

**Table 2:** Pharmacokinetic parameters of chlorzoxazone in rat plasma after X-ray irradiation.

Parameters	0 Gy	1 Gy	5 Gy
K_e_, 1/h	0.27 ± 0.05	0.31 ± 0.03	0.33 ± 0.007
t_1/2_, h	2.69 ± 0.595	2.23 ± 0.24	2.20 ± 0.58
MRT_(0-12)_, h	2.62 ± 0.19	2.49 ± 0.19	2.46 ± 0.23
CL, L/kg/h	0.84 ± 0.13	1.21 ± 0.30^*^	1.60 ± 0.52^**#^
Vd, L/kg	3.25 ± 0.72	3.96 ± 1.37	5.42 ± 3.16
AUC_(0-12)_, h·μg/ml	114.35 ± 17.39	80.38 ± 20.85**	63.97 ± 23.80^**^
AUC_(0-∞)_, h·μg/ml	121.41 ± 18.30	86.94 ± 21.32**	68.91 ± 23.10^**^
AUMC_(0-12)_, h^2^·μg/ml	299.58 ± 48.54	199.51 ± 51.61**	152.83 ± 45.55^**#^
AUMC_(0-∞)_, h^2^·μg/ml	407.69 ± 61.31	279.65 ± 55.40**	213.61 ± 36.40^**##^
T_max_, h	0.56 ± 0.26	0.68 ± 0.28	0.68 ± 0.28
C_max_, μg/ml	39.37 ± 5.67	26.49 ± 6.44^**^	20.43 ± 8.98^**#^

Data are expressed as the mean ± SD values (N = 10). *P < 0.05, **P < 0.01 compared to the 0 Gy group; ^#^ *P* < 0.05, ^##^ *P* < 0.01 compared to the 1 Gy group.

ke, elimination rate constant; t1/2, half-life; CL, total plasma clearance; Vd, apparent volume of distribution; Cmax, the maximum plasma concentration; AUC, area under the concentration time curve; AUMC, AUC of the first moment; MRT, mean residence time.

Relative to the value in the 0 Gy group, AUC_0-10_ values for chlorzoxazone were 1.4- and 1.8-fold lower in the 1 Gy and 5 Gy groups (both *P* < 0.01), respectively. CL values were 1.4- (P < 0.05) and 1.9- (P < 0.01) fold higher in the 1 Gy and 5 Gy groups, respectively, than in the 0 Gy group and 1.3-fold higher in the 5 Gy group than in the 1 Gy group (*P* < 0.05). C_max_ values were 32.7% and 48.1% lower in the 1 Gy and 5 Gy groups (both *P* < 0.01), respectively, compared to the 0 Gy group, and 22.9% lower in the 5 Gy group than in the 1 Gy group (*P* < 0.05). When comparing the 1 Gy and 5 Gy groups to the 0 Gy group, no statistically significant change in Vd was observed due to interindividual variations. However, it tended to enlarge with increasing irradiation and was 21.8% and 66.8% higher in the 1 Gy and 5 Gy groups, respectively, compared to the 0 Gy group. For t_1/2_, MRT, K_e_, and T_max_ values, we found no significant differences when comparing the 1 Gy and 5 Gy groups to the 0 Gy group.

The metabolism of caffeine and *chlorzoxazone* increases under X-ray irradiation as CL levels increase and AUC levels decrease, suggesting that CYP1A2 and CYP2E1 activity is enhanced in rats after X-ray irradiation.

### Protein Expression of CYP1A2 and CYP2E1

**Figure 6:**
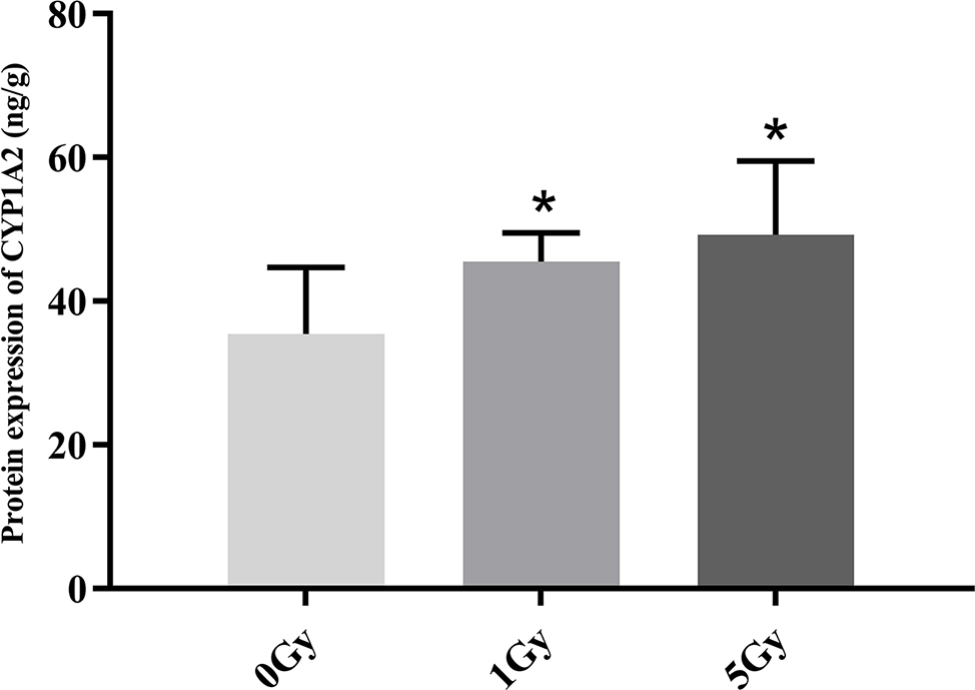
Protein expression of CYP1A2 in rats after exposure to X-ray irradiation. The data are presented as mean ± SD. N = 10. The data were analyzed using ANOVA, and the differences between the means of two groups were compared using LSD tests. *P < 0.05 compared to the 0 Gy group.

**Figure 7:**
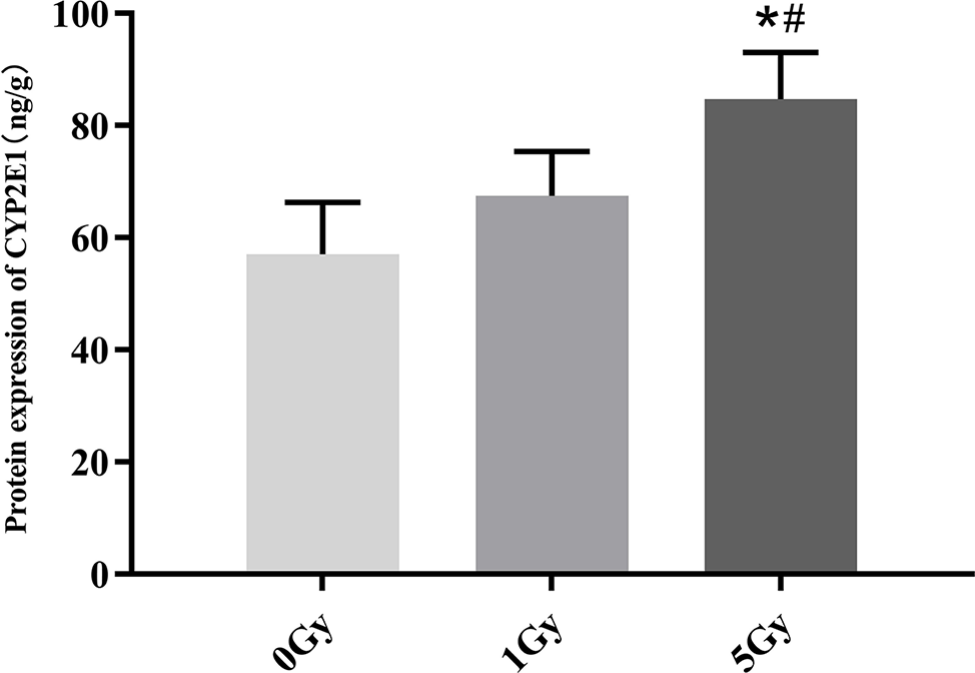
Protein expression of CYP2E1 in rats after exposure to X-ray irradiation. The data are presented as mean ± SD. N = 10. The data were analyzed using ANOVA, and the differences between the means of two groups were compared using LSD tests. *P < 0.05 compared to the 0 Gy group; ^#^ *P* < 0.05 compared to the 1 Gy group.

CYP1A2 and CYP2E1 protein expression levels were enhanced significantly by X-ray irradiation in rats. **Figures 6** and **7** show changes in the protein expression levels of CYP1A2 and CYP2E1 in rats after exposure to X-ray irradiation, respectively. Relative to the 0 Gy group, CYP1A2 protein expression levels were 28.3% and 38.9% higher for the 1 Gy and 5 Gy groups (both *P* < 0.05), respectively. The CYP2E1 protein expression levels were 48.4% higher for the 5 Gy group relative to those for the 0 Gy group (*P* < 0.05) and 25.6% higher for the 5 Gy group relative to those for the 1 Gy group (*P* < 0.05).

### mRNA Expression of CYP1A2 and CYP2E1

**Figure 8:**
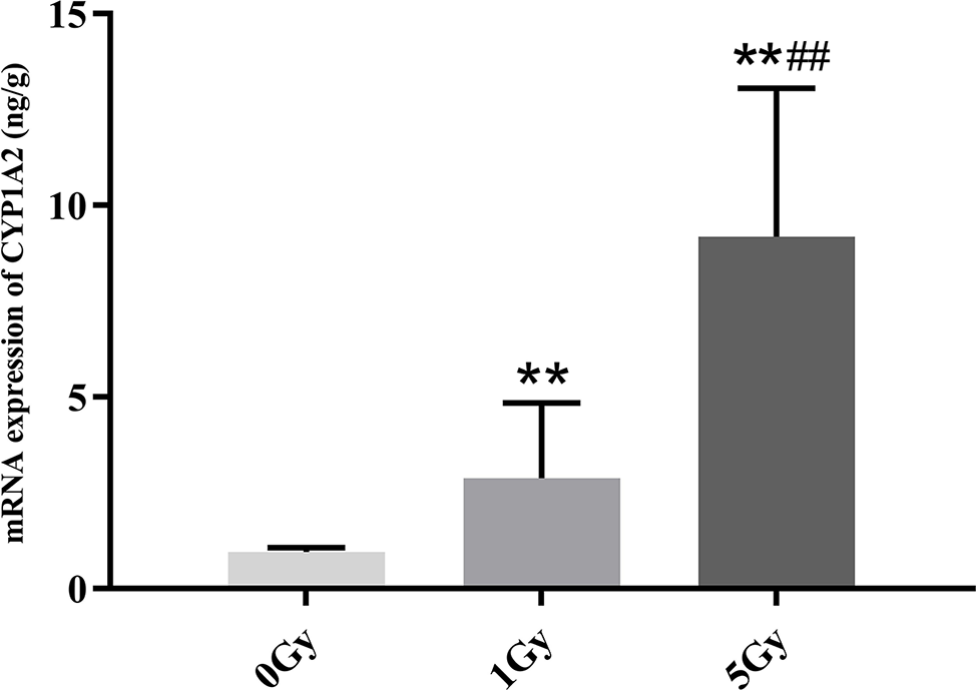
The mRNA expression of CYP1A2 in rats after exposure to X-ray irradiation. The data are presented as mean ± SD. N = 10. The data were analyzed using ANOVA, and the differences between the means of two groups were compared using LSD tests. **P < 0.01 compared to the 0 Gy group; ^##^ *P* < 0.01 compared to the 1 Gy group.

**Figure 9:**
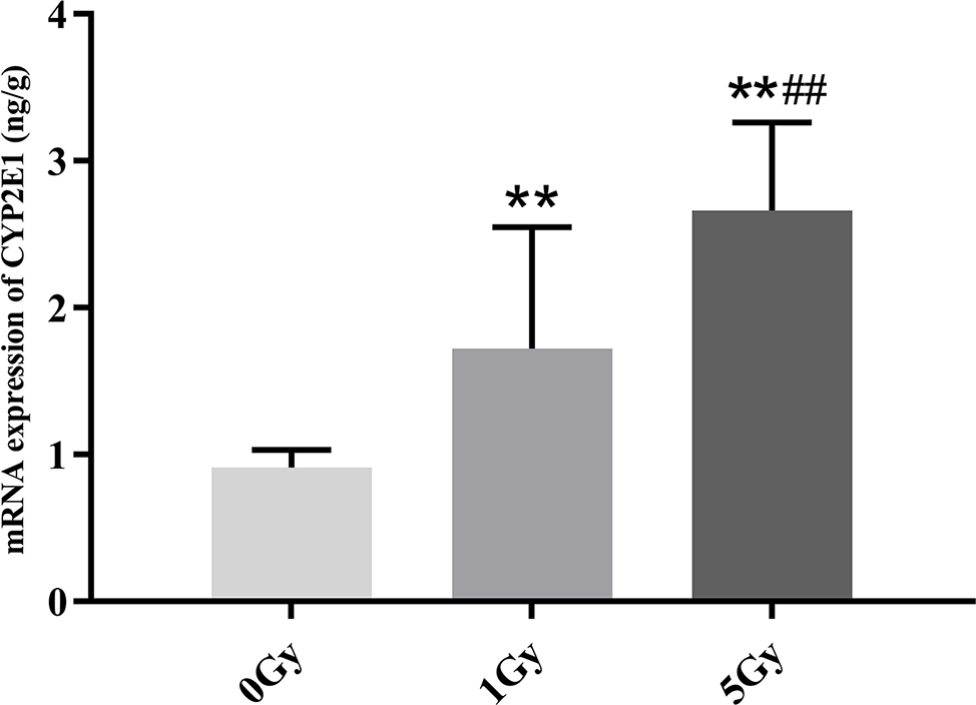
The mRNA expression of CYP2E1 in rats after exposure to X-ray irradiation. The data are presented as mean ± SD. N = 10. The data were analyzed using ANOVA, and the differences between the means of two groups were compared using LSD tests. **P < 0.01 compared to the 0 Gy group; ^##^ *P* < 0.01 compared to the 1 Gy group.

CYP1A2 and CYP2E1 mRNA expression levels were enhanced significantly by X-ray irradiation in rats. **Figures 8** and **9** show changes in the mRNA expression levels of CYP1A2 and CYP2E1 in rats after exposure to X-ray irradiation, respectively. The mRNA expression level of CYP1A2 was 200.0% and 856.3% higher for the 1 Gy and 5 Gy groups (both *P* < 0.01), respectively, than for the 0 Gy group and 218.8% higher for the 5 Gy group than for the 1 Gy group (*P* < 0.01). CYP2E1 mRNA expression level was 89.0% and 192.3% higher for the 1 Gy and 5 Gy groups (both *P* < 0.01), respectively, than for the 0 Gy group and 54.7% higher for the 5 Gy group relative to the 1 Gy group (*P* < 0.01).

## Discussion

A cocktail method was used to evaluate CYP1A2 and CYP2E1 activity. CYP450 activity can be determined from the ratio of probe drugs and their metabolites in the plasma or serum at a certain time point after probe drugs are administered or from changes in the pharmacokinetic parameters of probe substrates (Li et al., 2014a; Wu et al., 2015). Due to differences observed among individuals, there are some limitations in evaluating CYP450 activity from the ratio of probe drugs and their metabolites at a certain time point. For this study, caffeine and chlorzoxazone were selected as probe drugs for CYP1A2 and CYP2E1, respectively, and CYP1A2 and CYP2E1 activity was evaluated from pharmacokinetic changes in the probes. In addition, drug doses, the timing of blood collection, and analytical methods were optimized according to the relevant literature. In our pre-experiment, the pharmacokinetic study and tissue samplings were conducted around 0 and 24 hours following irradiation, and the results showed a slight significant difference between the irradiation and control groups. In this study, therefore, the pharmacokinetic experiments and tissue samplings were conducted around 40 hours following irradiation.

The present study is the first to investigate the activity and expression levels of CYP1A2 and CYP2E1 in rats after exposure to X-ray irradiation. γ-ray irradiation from Co^60^ has been used as modeling material in previous studies (Hsieh et al., 2011; Tsai et al., 2015), but the use of Co^60^ has been gradually discontinued due to its harmful effects on patients. Linear accelerators customize electrons or high-energy X-rays to conform to a tumor's shape and to destroy cancer cells while sparing the surrounding normal tissue, and they are now commonly used in radiotherapy due to their reliability, wide energy wavelength, and user-friendly interface (Janatpour et al., 2005; Thwaites and Tuohy, 2006; Griffiths et al., 2007). In our study, therefore, rat models were established with a one-time X-ray generated from a linear accelerator and applied to the whole body.

The body's hematopoietic and immune systems are the most sensitive to ionizing irradiation (Sanzari et al., 2013). Irradiation can inhibit or destroy the proliferation of the hematopoietic stem cells, granulocyte progenitors, and erythroid progenitor cells, and it can cause the dysfunction of the bone marrow hematopoietic system, the destruction of hematopoietic microenvironments, microcirculation disturbances, and the decline of peripheral blood cell counts (Volpi and Tarugi, 1999). Previous studies have shown that the number of white blood cells and the thymus and spleen indexes are decreased significantly in mice after exposure to X-ray irradiation (Duan et al., 2015). Our results show that white blood cell counts were 26.0% and 61.3% lower, that thymus indexes were 41.8% and 72.8% lower, and that spleen indexes were 19.9% and 56.8% lower in rats after X-ray irradiation at 1 Gy and 5 Gy, respectively. The thymus and spleen are the two main immune organs, and the thymus and spleen indexes can reflect and predict the immune function of the body. In our study, the indexes of the thymus and spleen were significantly decreased after irradiation, which indicated atrophy of the thymus and spleen. This may be mainly due to the increased apoptosis of the thymus and spleen cells after irradiation. Under the combined action of the first and second signals of antigen stimulation, a series of specific and non-specific immune responses are generated. This long-lasting, strong response further aggravates immune organ damage (Cao et al., 2004). In addition, the physiologic changes produced by irradiation are extremely complex and not completely understood. Total protein levels were higher in the irradiation groups than in the control group in the current study. Whether the protein binding rate of caffeine and chlorzoxazone changes under X-ray irradiation and further affects their pharmacokinetics needs further evaluation.

The activity of CYP1A2 and CYP2E1 was increased significantly in rats after exposure to X-ray irradiation. We found statistically significant differences in several pharmacokinetic parameters of caffeine and chlorzoxazone obtained from plasma data in rats after exposure to X-ray irradiation. Compared with the 0 Gy group, whole-body irradiation decreased the AUC of caffeine and chlorzoxazone and increased the CL. With respect to pharmacokinetics, this suggests that X-ray irradiation could facilitate the excretion of caffeine and chlorzoxazone. The decrease in AUC observed after 1 Gy or 5 Gy exposure to X-ray irradiation is consistent with the observed CL. When comparing the 1 Gy and 5 Gy groups to the 0 Gy group, no statistically significant change in the Vd of chlorzoxazone was observed due to interindividual variations. However, this parameter tended to enlarge with increasing irradiation. Mean plasma concentration–time profiles for chlorzoxazone in the treated groups seem to just shift parallely downward, which may indicate that Vd is the only factor needed to describe the effect of irradiation. The pharmacokinetic parameters of T_max_ of caffeine and K_e_, t_1/2_, MRT as well as T_max_ of chlorzoxazone obtained from the 1 Gy and 5 Gy groups were in accord with those found in the 0 Gy group. Our results are reminiscent of the effect of irradiation on the pharmacokinetics of 5-fluorouracil and irinotecan hydrochloride reported in recent studies. Whole pelvic and abdominal γ-ray irradiation with 2 Gy decreased the AUC of 5-fluorouracil in rats by 21.5% and 31.7%, respectively (Hsieh et al., 2010; Hsieh et al., 2011; Hsieh et al., 2015). Our previous study indicated that whole-body X-ray irradiation decreased the AUC of irinotecan hydrochloride and increased the CL (Qiao et al., 2019).

We found statistically significant increases in the activity and expression levels of CYP1A2 and CYP2E1 in rats after exposure to X-ray irradiation, and the changes in the activity observed echo changes in protein and mRNA expression levels. Previous studies show that successive exposure to γ-ray irradiation at 0.5 and 1 Gy does not affect the expression of CYP2E1, though protein and mRNA expression levels increase 3.6- and 2.5-fold after successive exposure to 3 Gy γ-ray (Chung et al., 2001; Maksymchuk et al., 2008). Chung et al. assessed the expression of CYP1A2 and CYP3A in rats after γ-ray irradiation, and they found that 3 Gy γ-rays do not change the expression levels of CYP1A2 and CYP3A (Chung et al., 2001). Ahn et al. found that the expression level of CYP2E1 exhibited a significant 2.3-fold increase in rats after uranium irradiation (Ahn et al., 2003). Lee et al. and Chung et al. investigated the effects of uranium irradiation on CYP1A2, CYP2E1, and CYP3A1, and their results showed that the expression level of CYP1A2 remained unchanged by uranium radiation, whereas the levels for CYP2E1 and CYP3A1 exhibited 2–4-fold and 4-fold increases, respectively (Chung et al., 2003; Lee et al., 2006). We found that CES1 and CYP3A1 protein and mRNA expression levels increased significantly in rats after exposure to X-ray irradiation. The irradiation-CYP450 phenomena must be therefore further investigated (Qiao et al., 2019).

Given that the concurrent use of chemotherapeutics in combination with radiotherapy is improving clinical treatment outcomes for an increasing number of malignancies (Herskovic et al., 1992; Eifel et al., 2004), the present study is timely. We show that X-ray irradiation can affect CYP1A2 and CYP2E1 activity and expression levels, which provides a reason for considering adjustments to chemotherapeutic administration during radiotherapy. The effect of these changes on the pharmacokinetics of drugs will depend upon the isoform involved in their biotransformation. We may predict that the AUC for substrates of CYP1A2 and CYP2E1, such as tamoxifen, pomalidomide, and etoposide, should be lower due to the decreased absorption and increased first pass and renal clearance levels under X-ray irradiation. The effect of X-ray irradiation on the systemic pharmacokinetics of chemotherapeutic agents or on other drugs is clearly in need of further clinical evaluation. Our study was performed on healthy rats. It is not known whether the same results would be observed in humans. Also, the significance of these changes may be different for patient populations, children, or the elderly. Moreover, theobromine, theophylline, and paraxanthine for caffeine and 6-hydroxy chlorzoxazone for chlorzoxazone should be measured simultaneously to support our findings. Given the significant changes in the absorption of caffeine and chlorzoxazone, one can speculate that intestinal CYP induction took place as well under irradiation. In addition, the pharmacokinetics of oral anti-cancer drugs, such as kinase inhibitors, is influenced by the intestinal CYP System. Additional experiments are required to further evaluate the activity and expression of intestinal CYP1A2 and CYP2E1 under X-ray irradiation.

## Conclusion

The present study is the first to investigate the activity and expression levels of CYP1A2 and CYP2E1 in rats after exposure to X-ray irradiation. This study reveals significant increases in the activity and expression levels of CYP1A2 and CYP2E1 in rats after exposure to X-ray irradiation, and the changes in activity observed echo changes in protein and mRNA expression levels. These findings support the suggestion that the pharmacokinetics of drugs during concurrent chemoradiation therapy should be re-checked and the optimal dose should be reevaluated, and adjusted if necessary, during concurrent chemoradiation therapy.

## Data Availability Statement

The datasets generated for this study are available on request to the corresponding author.

## Ethics Statement

The experimental procedures involving animals in this study were approved by the Animal Ethics Committee of the Medical College of Qinghai University. Experimental protocols were followed with strict adherence to the regulations set forth by the Experimental Animal Regulation by the National Science and Technology Commission, China, for the use of laboratory animals.

## Author Contributions

X-YL and NQ conceived and designed the research study. Y-YX, J-BZ, and J-XY participated in the acquisition of samples and data. XB and Y-BD analyzed and interpreted the data. X-JW, X-YL, NQ, and Y-BD wrote and revised the manuscript. All authors listed have made substantial, direct, and intellectual contributions to the work, and all approved it for publication.

## Funding

This study was supported by the National Natural Science Fund of China (No. 81760673 and 81660197) and the Natural Science Fund of Qinghai province, China (No. 2019-ZJ-918).

## Conflict of Interest

The authors declare that the research was conducted in the absence of any commercial or financial relationships that could be construed as a potential conflict of interest.
