# Supplementary material for: Regulation of X-Ray Irradiation on the Activity and Expression Levels of CYP1A2 and CYP2E1 in Rats
Source: Front Pharmacol. 2020 Jan 28;10:1575. doi: 10.3389/fphar.2019.01575 (PMC6997339; doi:10.3389/fphar.2019.01575)
Supplement: Supplementary file 1 [file DataSheet_1.pdf]

Supplementary Data

Regulation of X-Ray Irradiation on the Activity and Expression Levels of CYP1A2 and CYP2E1 in Rats

Xiang-Yang Li[1,2†], Ning Qu[3†], Xue-Jun Wang[4], Jian-Xin Yang[2], Yuan-Yao Xin[5], Jun-Bo Zhu[2], Xue Bai[2]and Ya-Bin Duan[2]*

[1]State Key Laboratory of Plateau Ecology and Agriculture, Qinghai University, Xining, China, [2]Medical College, Qinghai University, Xining, China, [3] Department of Anesthesiology, Qinghai Hospital of Traditional Chinese Medicine, Xining, China, [4]Department of Anesthesiology, Red Cross Hospital of Qinghai, Xining, China, [5]College of Eco-Environmental Engineering, Qinghai University, Xining, China

Supplemental contains:

Figure S1. A practical device for holding rats and medical electronic linear accelerator.

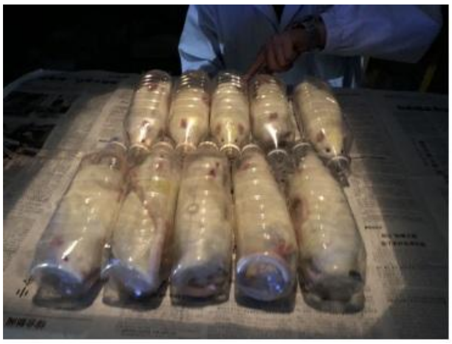

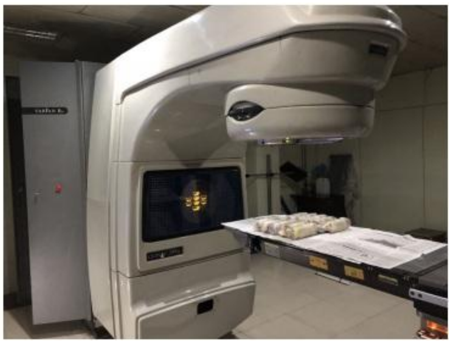

Figure S2. Total RNA tested by gel electrophoresis. M=2000 bp marker.

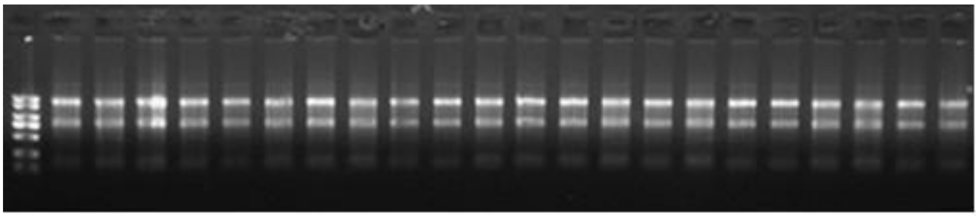

Figure S3. Amplification curves of SYBR green-based real time qPCR assay(A). Melt curve(B). Melt peak(C)

A

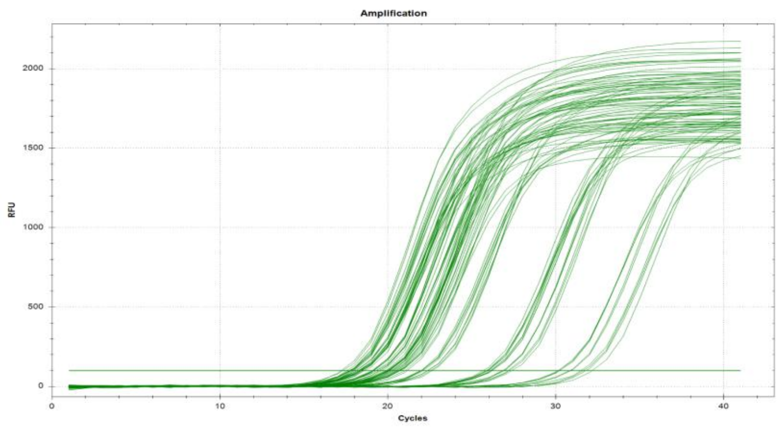
Amplification
RFU
2000
1500
1000
500
0
0
10
20
30
40
Cycles

B

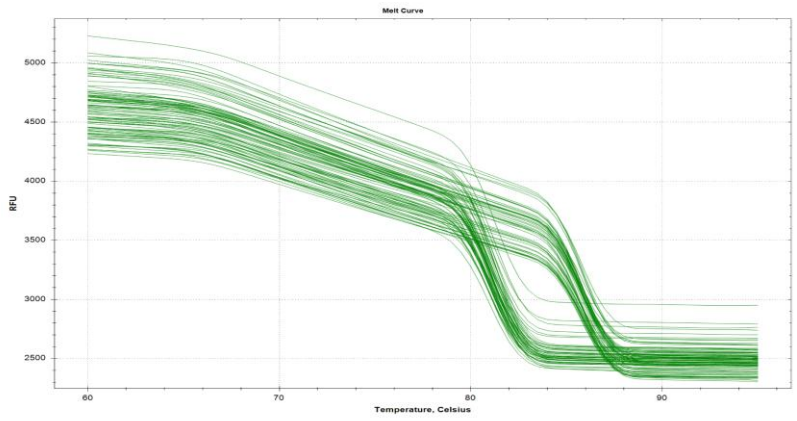
Melt Curve
RFU
5000
4500
4000
3500
3000
2500
60
70
80
90
Temperature, Celsius

C

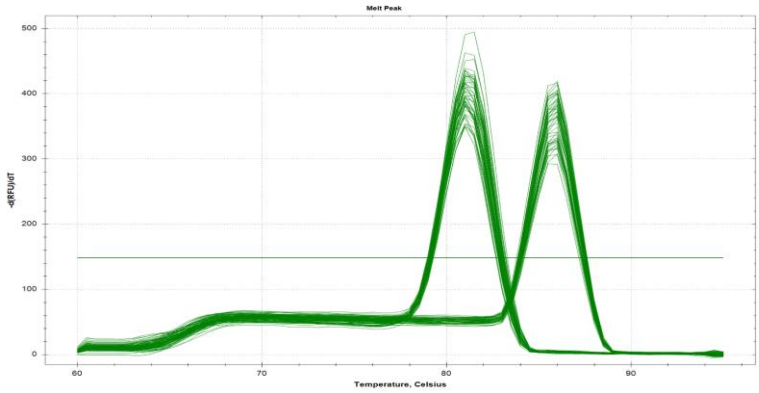
Melt Peak
-d(RFU)/dT
500
400
300
200
100
0
60
70
80
90
Temperature, Celsius
